# Bis{2-[2-(isopropyl­ammonio)ethyl­imino­meth­yl]-5-methoxy­phenolato}copper(II) bis­(perchlorate)

**DOI:** 10.1107/S1600536810017472

**Published:** 2010-05-19

**Authors:** Chen-Yi Wang, Feng Cao, Ping Wang, Xiang Wu, Cai-Jun Yuan

**Affiliations:** a Department of Chemistry, Huzhou University, Huzhou 313000, People’s Republic of China; b College of Chemical Engineering, Nanjing Forestry University, Nangjing 210037, People’s Republic of China

## Abstract

In the title compound, [Cu(C_13_H_20_N_2_O_2_)_2_](ClO_4_)_2_, the Cu^II^ atom in the complex dication is chelated by two phenolate O atoms and two imine N atoms from two zwitterionic 2-[2-(isopropyl­ammonio)ethyl­imino­meth­yl]-5-methoxy­phenolate ligands, forming a distorted square-planar geometry. One of the perchlorate anions is disordered over two sites with occupancies of 0.611 (15) and 0.389 (15). Intra­molecular N—H⋯O hydrogen bonds are observed in the complex dication. In the crystal structure, the perchlorate anions are linked to complex dications by inter­molecular N—H⋯O hydrogen bonds.

## Related literature

For general background to Cu^II^ complexes, see: Collinson & Fenton (1996 ▶); Hossain *et al.* (1996 ▶); Tarafder *et al.* (2002 ▶); Musie *et al.* (2003 ▶); García-Raso *et al.* (2003 ▶); Reddy *et al.* (2000 ▶); Ray *et al.* (2003 ▶); Arnold *et al.* (2003 ▶); Raptopoulou *et al.* (1998 ▶). For related structures, see: Wang *et al.* (2009*a* ▶,*b* ▶, 2010 ▶); Wang (2009 ▶). For bond lengths and angles in related Cu^II^ complexes, see: Hebbachi & Benali-Cherif (2005 ▶); Butcher *et al.* (2003 ▶); Elmali *et al.* (2000 ▶); Warda *et al.* (1997 ▶).
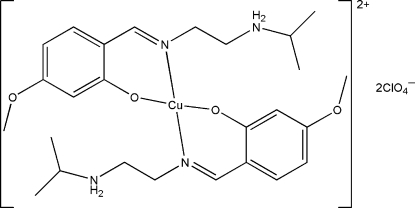


## Experimental

### 

#### Crystal data

[Cu(C_13_H_20_N_2_O_2_)_2_](ClO_4_)_2_
*M* *_r_* = 735.06
Orthorhombic, 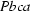

*a* = 17.4415 (13) Å
*b* = 14.009 (1) Å
*c* = 26.350 (2) Å
*V* = 6438.2 (8) Å^3^
*Z* = 8
Mo *K*α radiation
μ = 0.91 mm^−1^
*T* = 298 K
0.20 × 0.18 × 0.17 mm

#### Data collection

Bruker SMART CCD area-detector diffractometer
Absorption correction: multi-scan (*SADABS*; Sheldrick, 1996 ▶) *T*_min_ = 0.839, *T*_max_ = 0.861
36964 measured reflections
7004 independent reflections
3260 reflections with *I* > 2σ(*I*)
*R*_int_ = 0.117

#### Refinement

*R*[*F* ^2^ > 2σ(*F* ^2^)] = 0.059
*wR*(*F* ^2^) = 0.182
*S* = 1.01
7004 reflections
449 parameters
94 restraints
H-atom parameters constrained
Δρ_max_ = 0.67 e Å^−3^
Δρ_min_ = −0.39 e Å^−3^

### 

Data collection: *SMART* (Bruker, 1998 ▶); cell refinement: *SAINT* (Bruker, 1998 ▶); data reduction: *SAINT*; program(s) used to solve structure: *SHELXS97* (Sheldrick, 2008 ▶); program(s) used to refine structure: *SHELXL97* (Sheldrick, 2008 ▶); molecular graphics: *SHELXTL* (Sheldrick, 2008 ▶); software used to prepare material for publication: *SHELXTL*.

## Supplementary Material

Crystal structure: contains datablocks global, I. DOI: 10.1107/S1600536810017472/ci5083sup1.cif

Structure factors: contains datablocks I. DOI: 10.1107/S1600536810017472/ci5083Isup2.hkl

Additional supplementary materials:  crystallographic information; 3D view; checkCIF report

## Figures and Tables

**Table 1:** Selected bond lengths (Å)

Cu1—O1	1.925 (3)
Cu1—O3	1.933 (3)
Cu1—N3	1.969 (4)
Cu1—N1	1.970 (4)

**Table 2:** Hydrogen-bond geometry (Å, °)

*D*—H⋯*A*	*D*—H	H⋯*A*	*D*⋯*A*	*D*—H⋯*A*
N4—H4*B*⋯O1	0.90	1.86	2.705 (5)	156
N4—H4*A*⋯O12	0.90	2.04	2.930 (13)	171
N2—H2*B*⋯O3	0.90	2.23	2.849 (5)	125
N2—H2*A*⋯O6	0.90	2.20	3.070 (9)	163

**Fig. 1.**
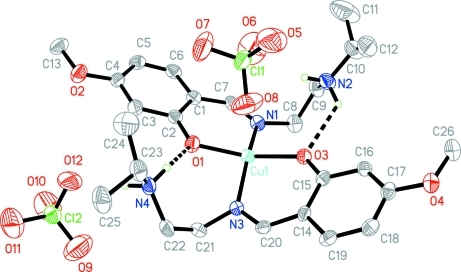
The structure of the title complex, showing the atom-numbering scheme. Displacement ellipsoids are drawn at the 30% probability level. C-bound H atoms have been omitted for clarity. Only the major disorder component of one of the perchlorate anions is shown.

**Fig. 2.**
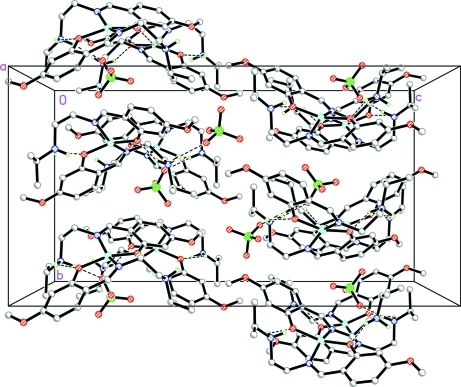
The crystal packing of the title compound. Intermolecular N—H···O hydrogen bonds are drawn as dashed lines.

## supplementary crystallographic information

### Comment

In recent years, copper(II) complexes have received much attention for their interesting biological activities and versatile structures (Collinson & Fenton, 1996; Hossain *et al.*, 1996; Tarafder *et al.*, 2002; Musie *et al.*, 2003; García-Raso *et al.*, 2003). Considerable effort has been made to construct a variety of copper(II) complexes in an attempt to model the physical and chemical behaviour of copper-containing metalloenzymes (Reddy *et al.*, 2000). The peculiarity of copper lies in its ability to form complexes with coordination number four, five, and six (Ray *et al.*, 2003; Arnold *et al.*, 2003; Raptopoulou *et al.*, 1998). As part of our investigations into novel urease inhibitors (Wang *et al.*, 2009a,b,2010; Wang, 2009), the title compound, a new Cu^II^ complex, has been synthesized, and its crystal structure is reported here.

The asymmetric units contains one mononuclear copper(II) complex dication, and two perchlorate anions (Fig. 1). The Cu^II^ atom in the dication is chelated by two phenolate O atoms and two imine N atoms from two 2-[(2-isopropylammonioethylimino)methyl]-5-methoxyphenolate ligands, forming a square-planar geometry. The coordinate bond lengths (Table 1) and angles are typical and are comparable with those observed in other related copper(II) complexes (Hebbachi & Benali-Cherif, 2005; Butcher *et al.*, 2003; Elmali *et al.*, 2000; Warda *et al.*, 1997). There are two intramolecular N—H···O hydrogen bonds in the complex dication.

In the crystal structure, the perchlorate anions are linked to the complex dications by intermolecular N—H···O hydrogen bonds (Table 2 and Fig. 2).

### Experimental

4-Methoxysalicylaldehyde (1.0 mmol, 152 mg), *N*-isopropyl-1,2-diaminoethane (1.0 mmol, 102 mg) and Cu(ClO_4_)_2_.6H_2_O (1.0 mmol, 370 mg) were dissolved in methanol (80 ml). The mixture was stirred at room temperature for about 1 h to give a blue solution. After keeping the solution in air for a few days, blue block-like crystals were formed.

### Refinement

H atoms were placed in geometrically idealized positions and constrained to ride on their parent atoms, with C–H distances of 0.93–0.98 Å, N—H distances of 0.90 Å, and with U_iso_(H) set at 1.2U_eq_(C,N) and 1.5U_eq_(C_methyl_). One of the perchlorate anions is disordered over two distinct sites with occupancies of 0.611 (15) and 0.389 (15). The positional and U^ij^ parameters of disordered atoms Cl2 and Cl2' were constrained to be the same. The Cl···O and O···O distances in the disorder components were restrained to 1.42 (1) and 2.35 (2) Å, respectively. The U^ij^ parameters of disordered O atoms were restrained to an approximate isotropic behaviour. The C10—C11 and C10—C12 distances were restrained to 1.540 (8) Å.

### Crystal data

**Table:**

[Cu(C_13_H_20_N_2_O_2_)_2_](ClO_4_)_2_	*F*(000) = 3064
*M**_r_* = 735.06	*D*_x_ = 1.517 Mg m^−^^3^
Orthorhombic, *P**b**c**a*	Mo *K*α radiation, λ = 0.71073 Å
Hall symbol: -P 2ac 2ab	Cell parameters from 2387 reflections
*a* = 17.4415 (13) Å	θ = 2.4–24.5°
*b* = 14.009 (1) Å	µ = 0.91 mm^−^^1^
*c* = 26.350 (2) Å	*T* = 298 K
*V* = 6438.2 (8) Å^3^	Block, blue
*Z* = 8	0.20 × 0.18 × 0.17 mm

### Data collection

**Table:**

Bruker SMART CCD area-detector diffractometer	7004 independent reflections
Radiation source: fine-focus sealed tube	3260 reflections with *I* > 2σ(*I*)
graphite	*R*_int_ = 0.117
ω scan	θ_max_ = 27.0°, θ_min_ = 1.6°
Absorption correction: multi-scan (*SADABS*; Sheldrick, 1996)	*h* = −22→20
*T*_min_ = 0.839, *T*_max_ = 0.861	*k* = −17→17
36964 measured reflections	*l* = −29→33

### Refinement

**Table:**

Refinement on *F*^2^	Primary atom site location: structure-invariant direct methods
Least-squares matrix: full	Secondary atom site location: difference Fourier map
*R*[*F*^2^ > 2σ(*F*^2^)] = 0.059	Hydrogen site location: inferred from neighbouring sites
*wR*(*F*^2^) = 0.182	H-atom parameters constrained
*S* = 1.01	*w* = 1/[σ^2^(*F*_o_^2^) + (0.0802*P*)^2^ + 0.2786*P*] where *P* = (*F*_o_^2^ + 2*F*_c_^2^)/3
7004 reflections	(Δ/σ)_max_ = 0.001
449 parameters	Δρ_max_ = 0.67 e Å^−^^3^
94 restraints	Δρ_min_ = −0.38 e Å^−^^3^

### Special details

**Table:**

Geometry. All esds (except the esd in the dihedral angle between two l.s. planes) are estimated using the full covariance matrix. The cell esds are taken into account individually in the estimation of esds in distances, angles and torsion angles; correlations between esds in cell parameters are only used when they are defined by crystal symmetry. An approximate (isotropic) treatment of cell esds is used for estimating esds involving l.s. planes.
Refinement. Refinement of *F*^2^ against ALL reflections. The weighted *R*-factor wR and goodness of fit *S* are based on *F*^2^, conventional *R*-factors *R* are based on *F*, with *F* set to zero for negative *F*^2^. The threshold expression of *F*^2^ > σ(*F*^2^) is used only for calculating *R*-factors(gt) etc. and is not relevant to the choice of reflections for refinement. *R*-factors based on *F*^2^ are statistically about twice as large as those based on *F*, and *R*- factors based on ALL data will be even larger.

### Fractional atomic coordinates and isotropic or equivalent isotropic displacement parameters (Å^2^)

**Table:**

	*x*	*y*	*z*	*U*_iso_*/*U*_eq_	Occ. (<1)
Cu1	0.20772 (3)	0.30739 (5)	0.29436 (2)	0.0430 (2)	
Cl1	0.35617 (10)	0.50449 (12)	0.29759 (6)	0.0669 (5)	
N1	0.2829 (2)	0.2199 (3)	0.32521 (16)	0.0443 (11)	
N2	0.3178 (3)	0.3382 (3)	0.41567 (18)	0.0574 (13)	
H2A	0.3298	0.3618	0.3849	0.069*	
H2B	0.2676	0.3497	0.4207	0.069*	
N3	0.1097 (2)	0.3435 (3)	0.26267 (15)	0.0417 (10)	
N4	0.1766 (2)	0.4031 (3)	0.15898 (16)	0.0528 (12)	
H4A	0.1910	0.3676	0.1321	0.063*	
H4B	0.2056	0.3851	0.1856	0.063*	
O1	0.2543 (2)	0.2985 (3)	0.22831 (13)	0.0514 (10)	
O2	0.4443 (2)	0.2600 (3)	0.10520 (15)	0.0643 (11)	
O3	0.17867 (19)	0.3576 (2)	0.35993 (12)	0.0444 (9)	
O4	0.0587 (2)	0.5763 (3)	0.47332 (14)	0.0559 (10)	
O5	0.3648 (3)	0.5847 (5)	0.3286 (3)	0.147 (2)	
O6	0.3925 (4)	0.4248 (5)	0.3208 (3)	0.159 (3)	
O7	0.3923 (3)	0.5183 (5)	0.2529 (2)	0.148 (2)	
O8	0.2780 (3)	0.4867 (4)	0.2923 (3)	0.135 (2)	
Cl2'	0.17958 (9)	0.25224 (11)	0.03722 (6)	0.0601 (4)	0.389 (15)
O9'	0.1570 (12)	0.2204 (11)	0.0869 (4)	0.129 (7)	0.389 (15)
O10'	0.2039 (11)	0.1768 (11)	0.0069 (7)	0.122 (8)	0.389 (15)
O11'	0.1144 (9)	0.3020 (11)	0.0192 (8)	0.141 (8)	0.389 (15)
O12'	0.2391 (10)	0.3202 (14)	0.0434 (8)	0.132 (10)	0.389 (15)
Cl2	0.17958 (9)	0.25224 (11)	0.03722 (6)	0.0601 (4)	0.611 (15)
O9	0.1079 (6)	0.2451 (11)	0.0606 (6)	0.164 (6)	0.611 (15)
O10	0.2095 (8)	0.1581 (7)	0.0317 (6)	0.145 (6)	0.611 (15)
O11	0.1749 (9)	0.2871 (9)	−0.0129 (3)	0.157 (6)	0.611 (15)
O12	0.2292 (6)	0.3064 (10)	0.0667 (5)	0.133 (6)	0.611 (15)
C1	0.3633 (3)	0.2031 (3)	0.2500 (2)	0.0433 (13)	
C2	0.3208 (3)	0.2609 (4)	0.21648 (19)	0.0419 (12)	
C3	0.3518 (3)	0.2759 (4)	0.1675 (2)	0.0496 (14)	
H3	0.3243	0.3121	0.1441	0.060*	
C4	0.4217 (3)	0.2383 (4)	0.1535 (2)	0.0466 (13)	
C5	0.4627 (3)	0.1818 (4)	0.1865 (2)	0.0510 (14)	
H5	0.5096	0.1557	0.1769	0.061*	
C6	0.4335 (3)	0.1650 (4)	0.2330 (2)	0.0510 (15)	
H6	0.4611	0.1262	0.2550	0.061*	
C7	0.3393 (3)	0.1828 (4)	0.3006 (2)	0.0447 (13)	
H7	0.3678	0.1372	0.3180	0.054*	
C8	0.2723 (3)	0.1865 (4)	0.3781 (2)	0.0536 (15)	
H8A	0.2791	0.1178	0.3793	0.064*	
H8B	0.2205	0.2008	0.3890	0.064*	
C9	0.3286 (4)	0.2335 (4)	0.4139 (2)	0.0597 (16)	
H9A	0.3220	0.2073	0.4477	0.072*	
H9B	0.3805	0.2194	0.4029	0.072*	
C10	0.3603 (4)	0.3919 (5)	0.4532 (3)	0.084 (2)	
H10	0.3401	0.3683	0.4856	0.101*	
C11	0.4435 (4)	0.3724 (7)	0.4567 (4)	0.151 (4)	
H11A	0.4674	0.3862	0.4247	0.182*	
H11B	0.4515	0.3065	0.4651	0.182*	
H11C	0.4657	0.4120	0.4826	0.182*	
C12	0.3397 (4)	0.4960 (4)	0.4536 (3)	0.085 (2)	
H12A	0.3656	0.5271	0.4812	0.102*	
H12B	0.2853	0.5028	0.4578	0.102*	
H12C	0.3551	0.5247	0.4222	0.102*	
C13	0.5160 (3)	0.2259 (5)	0.0871 (3)	0.0691 (18)	
H13A	0.5554	0.2416	0.1111	0.083*	
H13B	0.5274	0.2552	0.0551	0.083*	
H13C	0.5136	0.1579	0.0830	0.083*	
C14	0.0646 (3)	0.4398 (4)	0.33394 (18)	0.0391 (12)	
C15	0.1234 (3)	0.4187 (4)	0.36975 (19)	0.0397 (12)	
C16	0.1200 (3)	0.4628 (4)	0.41700 (19)	0.0416 (13)	
H16	0.1566	0.4472	0.4413	0.050*	
C17	0.0644 (3)	0.5287 (4)	0.4289 (2)	0.0457 (13)	
C18	0.0072 (3)	0.5516 (4)	0.3937 (2)	0.0482 (14)	
H18	−0.0299	0.5971	0.4012	0.058*	
C19	0.0073 (3)	0.5055 (4)	0.3478 (2)	0.0472 (14)	
H19	−0.0321	0.5181	0.3250	0.057*	
C20	0.0594 (3)	0.3960 (4)	0.28523 (19)	0.0438 (13)	
H20	0.0140	0.4065	0.2675	0.053*	
C21	0.0873 (3)	0.3067 (4)	0.2121 (2)	0.0573 (16)	
H21A	0.0345	0.2853	0.2134	0.069*	
H21B	0.1191	0.2520	0.2039	0.069*	
C22	0.0957 (3)	0.3814 (5)	0.1706 (2)	0.0640 (18)	
H22A	0.0706	0.3586	0.1401	0.077*	
H22B	0.0701	0.4395	0.1812	0.077*	
C23	0.1938 (4)	0.5072 (4)	0.1473 (2)	0.0620 (17)	
H23	0.1742	0.5462	0.1753	0.074*	
C24	0.2780 (4)	0.5209 (5)	0.1442 (3)	0.093 (2)	
H24A	0.3018	0.4941	0.1738	0.112*	
H24B	0.2894	0.5878	0.1424	0.112*	
H24C	0.2973	0.4895	0.1144	0.112*	
C25	0.1517 (4)	0.5364 (5)	0.0988 (2)	0.082 (2)	
H25A	0.1738	0.5037	0.0703	0.098*	
H25B	0.1564	0.6041	0.0940	0.098*	
H25C	0.0985	0.5198	0.1017	0.098*	
C26	0.1108 (4)	0.5532 (4)	0.5135 (2)	0.0648 (17)	
H26A	0.1047	0.4873	0.5226	0.078*	
H26B	0.1001	0.5927	0.5424	0.078*	
H26C	0.1624	0.5640	0.5023	0.078*	

### Atomic displacement parameters (Å^2^)

**Table:**

	*U*^11^	*U*^22^	*U*^33^	*U*^12^	*U*^13^	*U*^23^
Cu1	0.0412 (4)	0.0492 (4)	0.0387 (4)	0.0057 (3)	−0.0002 (3)	0.0029 (3)
Cl1	0.0691 (11)	0.0669 (11)	0.0649 (11)	−0.0151 (9)	0.0239 (9)	−0.0104 (9)
N1	0.053 (3)	0.037 (2)	0.043 (3)	0.003 (2)	−0.005 (2)	0.005 (2)
N2	0.051 (3)	0.063 (3)	0.058 (3)	0.015 (2)	−0.012 (2)	−0.012 (2)
N3	0.039 (2)	0.049 (3)	0.037 (3)	−0.001 (2)	0.000 (2)	0.001 (2)
N4	0.051 (3)	0.074 (4)	0.034 (3)	0.017 (3)	−0.001 (2)	0.006 (2)
O1	0.046 (2)	0.064 (3)	0.044 (2)	0.019 (2)	0.0036 (18)	0.0074 (18)
O2	0.057 (3)	0.074 (3)	0.061 (3)	0.010 (2)	0.015 (2)	0.002 (2)
O3	0.041 (2)	0.051 (2)	0.041 (2)	0.0093 (18)	−0.0017 (16)	0.0019 (17)
O4	0.062 (3)	0.059 (3)	0.046 (2)	0.002 (2)	0.005 (2)	−0.0064 (19)
O5	0.115 (4)	0.140 (5)	0.186 (6)	−0.023 (4)	0.009 (4)	−0.075 (5)
O6	0.172 (6)	0.147 (5)	0.159 (6)	0.045 (5)	0.010 (5)	0.046 (5)
O7	0.124 (4)	0.236 (6)	0.083 (4)	0.000 (4)	0.044 (3)	0.022 (4)
O8	0.081 (4)	0.112 (4)	0.211 (6)	−0.031 (3)	0.039 (4)	−0.051 (4)
Cl2'	0.0687 (10)	0.0550 (10)	0.0566 (10)	−0.0030 (9)	−0.0040 (8)	0.0000 (8)
O9'	0.144 (11)	0.142 (10)	0.100 (9)	0.017 (8)	0.020 (8)	0.043 (8)
O10'	0.141 (11)	0.119 (11)	0.105 (10)	0.003 (8)	0.013 (8)	−0.047 (8)
O11'	0.116 (11)	0.145 (11)	0.161 (12)	0.028 (8)	−0.040 (9)	0.030 (8)
O12'	0.128 (12)	0.126 (12)	0.142 (13)	−0.047 (8)	0.004 (9)	−0.015 (9)
Cl2	0.0687 (10)	0.0550 (10)	0.0566 (10)	−0.0030 (9)	−0.0040 (8)	0.0000 (8)
O9	0.108 (8)	0.204 (10)	0.179 (10)	−0.006 (7)	0.050 (7)	−0.008 (8)
O10	0.188 (9)	0.102 (7)	0.144 (9)	0.044 (6)	−0.028 (8)	−0.011 (6)
O11	0.179 (10)	0.187 (10)	0.105 (8)	−0.003 (7)	−0.018 (7)	0.049 (6)
O12	0.082 (7)	0.208 (14)	0.109 (10)	−0.044 (7)	−0.002 (7)	−0.089 (9)
C1	0.043 (3)	0.039 (3)	0.047 (3)	0.005 (3)	−0.006 (3)	−0.003 (3)
C2	0.038 (3)	0.043 (3)	0.045 (3)	0.005 (3)	−0.003 (2)	−0.003 (2)
C3	0.046 (3)	0.057 (4)	0.046 (3)	0.014 (3)	−0.002 (3)	−0.002 (3)
C4	0.046 (3)	0.050 (3)	0.044 (3)	−0.007 (3)	0.005 (3)	−0.006 (3)
C5	0.039 (3)	0.050 (4)	0.064 (4)	0.007 (3)	0.002 (3)	−0.008 (3)
C6	0.045 (3)	0.052 (4)	0.056 (4)	0.013 (3)	−0.010 (3)	−0.004 (3)
C7	0.057 (3)	0.035 (3)	0.042 (3)	0.004 (3)	−0.011 (3)	0.000 (2)
C8	0.072 (4)	0.040 (3)	0.049 (3)	0.008 (3)	−0.001 (3)	0.011 (3)
C9	0.073 (4)	0.062 (4)	0.045 (3)	0.013 (3)	−0.009 (3)	0.007 (3)
C10	0.084 (5)	0.103 (6)	0.065 (5)	−0.010 (5)	−0.020 (4)	−0.003 (4)
C11	0.112 (6)	0.167 (8)	0.175 (8)	0.016 (6)	−0.071 (6)	−0.036 (6)
C12	0.094 (5)	0.077 (5)	0.084 (5)	−0.018 (4)	0.006 (4)	−0.024 (4)
C13	0.050 (4)	0.077 (5)	0.080 (5)	−0.003 (3)	0.021 (3)	−0.015 (4)
C14	0.038 (3)	0.046 (3)	0.033 (3)	0.002 (3)	0.007 (2)	0.004 (2)
C15	0.043 (3)	0.038 (3)	0.038 (3)	−0.003 (2)	0.006 (2)	0.009 (2)
C16	0.041 (3)	0.050 (3)	0.034 (3)	−0.004 (3)	−0.002 (2)	0.007 (2)
C17	0.053 (3)	0.042 (3)	0.042 (3)	−0.005 (3)	0.012 (3)	0.003 (3)
C18	0.043 (3)	0.044 (3)	0.058 (4)	0.008 (3)	0.008 (3)	0.001 (3)
C19	0.036 (3)	0.054 (4)	0.052 (4)	0.003 (3)	0.006 (3)	0.011 (3)
C20	0.032 (3)	0.059 (4)	0.041 (3)	−0.004 (3)	−0.002 (2)	0.013 (3)
C21	0.044 (3)	0.081 (4)	0.047 (4)	−0.006 (3)	−0.009 (3)	−0.006 (3)
C22	0.053 (4)	0.105 (5)	0.034 (3)	0.016 (4)	−0.010 (3)	0.002 (3)
C23	0.078 (5)	0.059 (4)	0.049 (4)	0.017 (4)	−0.003 (3)	−0.002 (3)
C24	0.095 (6)	0.078 (5)	0.107 (6)	−0.004 (4)	−0.007 (5)	0.019 (4)
C25	0.110 (6)	0.079 (5)	0.056 (4)	0.033 (4)	−0.004 (4)	0.012 (4)
C26	0.081 (5)	0.068 (4)	0.045 (4)	0.004 (4)	0.003 (3)	−0.008 (3)

### Geometric parameters (Å, °)

**Table:**

Cu1—O1	1.925 (3)	C8—H8B	0.97
Cu1—O3	1.933 (3)	C9—H9A	0.97
Cu1—N3	1.969 (4)	C9—H9B	0.97
Cu1—N1	1.970 (4)	C10—C11	1.479 (7)
Cl1—O7	1.349 (5)	C10—C12	1.502 (6)
Cl1—O8	1.393 (5)	C10—H10	0.98
Cl1—O5	1.398 (6)	C11—H11A	0.96
Cl1—O6	1.422 (6)	C11—H11B	0.96
N1—C7	1.287 (6)	C11—H11C	0.96
N1—C8	1.481 (6)	C12—H12A	0.96
N2—C10	1.447 (7)	C12—H12B	0.96
N2—C9	1.480 (7)	C12—H12C	0.96
N2—H2A	0.90	C13—H13A	0.96
N2—H2B	0.90	C13—H13B	0.96
N3—C20	1.290 (6)	C13—H13C	0.96
N3—C21	1.480 (6)	C14—C19	1.407 (7)
N4—C22	1.476 (7)	C14—C15	1.424 (7)
N4—C23	1.520 (7)	C14—C20	1.426 (7)
N4—H4A	0.90	C15—C16	1.391 (7)
N4—H4B	0.90	C16—C17	1.375 (7)
O1—C2	1.312 (6)	C16—H16	0.93
O2—C4	1.366 (6)	C17—C18	1.400 (7)
O2—C13	1.421 (6)	C18—C19	1.370 (7)
O3—C15	1.315 (6)	C18—H18	0.93
O4—C17	1.351 (6)	C19—H19	0.93
O4—C26	1.431 (6)	C20—H20	0.93
Cl2'—O10'	1.391 (8)	C21—C22	1.521 (8)
Cl2'—O11'	1.416 (8)	C21—H21A	0.97
Cl2'—O12'	1.418 (9)	C21—H21B	0.97
Cl2'—O9'	1.437 (8)	C22—H22A	0.97
C1—C2	1.409 (7)	C22—H22B	0.97
C1—C6	1.410 (7)	C23—C24	1.484 (8)
C1—C7	1.427 (7)	C23—C25	1.529 (8)
C2—C3	1.416 (7)	C23—H23	0.98
C3—C4	1.379 (7)	C24—H24A	0.96
C3—H3	0.93	C24—H24B	0.96
C4—C5	1.376 (7)	C24—H24C	0.96
C5—C6	1.348 (7)	C25—H25A	0.96
C5—H5	0.93	C25—H25B	0.96
C6—H6	0.93	C25—H25C	0.96
C7—H7	0.93	C26—H26A	0.96
C8—C9	1.513 (8)	C26—H26B	0.96
C8—H8A	0.97	C26—H26C	0.96
			
O1—Cu1—O3	160.46 (15)	C11—C10—H10	103.6
O1—Cu1—N3	89.97 (16)	C12—C10—H10	103.6
O3—Cu1—N3	93.31 (16)	C10—C11—H11A	109.5
O1—Cu1—N1	92.98 (16)	C10—C11—H11B	109.5
O3—Cu1—N1	91.84 (16)	H11A—C11—H11B	109.5
N3—Cu1—N1	155.90 (17)	C10—C11—H11C	109.5
O7—Cl1—O8	113.3 (4)	H11A—C11—H11C	109.5
O7—Cl1—O5	110.2 (5)	H11B—C11—H11C	109.5
O8—Cl1—O5	107.9 (4)	C10—C12—H12A	109.5
O7—Cl1—O6	106.2 (4)	C10—C12—H12B	109.5
O8—Cl1—O6	109.8 (4)	H12A—C12—H12B	109.5
O5—Cl1—O6	109.4 (5)	C10—C12—H12C	109.5
C7—N1—C8	116.1 (4)	H12A—C12—H12C	109.5
C7—N1—Cu1	123.5 (4)	H12B—C12—H12C	109.5
C8—N1—Cu1	120.1 (3)	O2—C13—H13A	109.5
C10—N2—C9	118.1 (5)	O2—C13—H13B	109.5
C10—N2—H2A	107.8	H13A—C13—H13B	109.5
C9—N2—H2A	107.8	O2—C13—H13C	109.5
C10—N2—H2B	107.8	H13A—C13—H13C	109.5
C9—N2—H2B	107.8	H13B—C13—H13C	109.5
H2A—N2—H2B	107.1	C19—C14—C15	118.4 (5)
C20—N3—C21	115.7 (4)	C19—C14—C20	118.0 (5)
C20—N3—Cu1	122.8 (3)	C15—C14—C20	123.6 (5)
C21—N3—Cu1	121.4 (3)	O3—C15—C16	119.7 (5)
C22—N4—C23	115.3 (5)	O3—C15—C14	122.2 (5)
C22—N4—H4A	108.4	C16—C15—C14	118.1 (5)
C23—N4—H4A	108.4	C17—C16—C15	122.1 (5)
C22—N4—H4B	108.4	C17—C16—H16	118.9
C23—N4—H4B	108.4	C15—C16—H16	118.9
H4A—N4—H4B	107.5	O4—C17—C16	125.5 (5)
C2—O1—Cu1	127.9 (3)	O4—C17—C18	114.1 (5)
C4—O2—C13	119.4 (5)	C16—C17—C18	120.4 (5)
C15—O3—Cu1	127.2 (3)	C19—C18—C17	118.4 (5)
C17—O4—C26	118.8 (4)	C19—C18—H18	120.8
O10'—Cl2'—O11'	115.3 (10)	C17—C18—H18	120.8
O10'—Cl2'—O12'	110.6 (10)	C18—C19—C14	122.5 (5)
O11'—Cl2'—O12'	107.2 (11)	C18—C19—H19	118.7
O10'—Cl2'—O9'	111.7 (10)	C14—C19—H19	118.7
O11'—Cl2'—O9'	103.8 (9)	N3—C20—C14	128.1 (5)
O12'—Cl2'—O9'	107.7 (10)	N3—C20—H20	116.0
C2—C1—C6	118.4 (5)	C14—C20—H20	116.0
C2—C1—C7	123.2 (5)	N3—C21—C22	112.4 (5)
C6—C1—C7	118.5 (5)	N3—C21—H21A	109.1
O1—C2—C1	123.1 (5)	C22—C21—H21A	109.1
O1—C2—C3	119.7 (5)	N3—C21—H21B	109.1
C1—C2—C3	117.2 (5)	C22—C21—H21B	109.1
C4—C3—C2	121.6 (5)	H21A—C21—H21B	107.8
C4—C3—H3	119.2	N4—C22—C21	112.6 (4)
C2—C3—H3	119.2	N4—C22—H22A	109.1
O2—C4—C5	124.5 (5)	C21—C22—H22A	109.1
O2—C4—C3	114.7 (5)	N4—C22—H22B	109.1
C5—C4—C3	120.7 (5)	C21—C22—H22B	109.1
C6—C5—C4	118.6 (5)	H22A—C22—H22B	107.8
C6—C5—H5	120.7	C24—C23—N4	109.2 (5)
C4—C5—H5	120.7	C24—C23—C25	113.2 (6)
C5—C6—C1	123.5 (5)	N4—C23—C25	109.3 (5)
C5—C6—H6	118.3	C24—C23—H23	108.3
C1—C6—H6	118.3	N4—C23—H23	108.3
N1—C7—C1	127.8 (5)	C25—C23—H23	108.3
N1—C7—H7	116.1	C23—C24—H24A	109.5
C1—C7—H7	116.1	C23—C24—H24B	109.5
N1—C8—C9	111.6 (5)	H24A—C24—H24B	109.5
N1—C8—H8A	109.3	C23—C24—H24C	109.5
C9—C8—H8A	109.3	H24A—C24—H24C	109.5
N1—C8—H8B	109.3	H24B—C24—H24C	109.5
C9—C8—H8B	109.3	C23—C25—H25A	109.5
H8A—C8—H8B	108.0	C23—C25—H25B	109.5
N2—C9—C8	111.6 (4)	H25A—C25—H25B	109.5
N2—C9—H9A	109.3	C23—C25—H25C	109.5
C8—C9—H9A	109.3	H25A—C25—H25C	109.5
N2—C9—H9B	109.3	H25B—C25—H25C	109.5
C8—C9—H9B	109.3	O4—C26—H26A	109.5
H9A—C9—H9B	108.0	O4—C26—H26B	109.5
N2—C10—C11	116.8 (6)	H26A—C26—H26B	109.5
N2—C10—C12	112.8 (5)	O4—C26—H26C	109.5
C11—C10—C12	114.4 (7)	H26A—C26—H26C	109.5
N2—C10—H10	103.6	H26B—C26—H26C	109.5

### Hydrogen-bond geometry (Å, °)

**Table:**

*D*—H···*A*	*D*—H	H···*A*	*D*···*A*	*D*—H···*A*
N4—H4B···O1	0.90	1.86	2.705 (5)	156
N4—H4A···O12	0.90	2.04	2.930 (13)	171
N2—H2B···O3	0.90	2.23	2.849 (5)	125
N2—H2A···O6	0.90	2.20	3.070 (9)	163
